# Radiological Assessment of Indoor Radon and Thoron Concentrations and Indoor Radon Map of Dwellings in Mashhad, Iran

**DOI:** 10.3390/ijerph18010141

**Published:** 2020-12-28

**Authors:** Mohammademad Adelikhah, Amin Shahrokhi, Morteza Imani, Stanislaw Chalupnik, Tibor Kovács

**Affiliations:** 1 Institute of Radiochemistry and Radioecology, University of Pannonia, 8200 Veszprém, Hungary; emad@mk.uni-pannon.hu (M.A.); ashahrokhi@almos.uni-pannon.hu (A.S.); 2 Materials and Nuclear Fuel Research School, Nuclear Science and Technology Research Institute, Tehran 11365-8486, Iran; imani.mrt@gmail.com; 3 Silesian Centre for Environmental Radioactivity, Central Mining Institute, plac Gwarków 1, 40-166 Katowice, Poland; schalupnik@gig.eu

**Keywords:** residential exposure, dose, gamma radiation, health risk, radon mapping, CR-39

## Abstract

A comprehensive study was carried out to measure indoor radon/thoron concentrations in 78 dwellings and soil-gas radon in the city of Mashhad, Iran during two seasons, using two common radon monitoring devices (NRPB and RADUET). In the winter, indoor radon concentrations measured between 75 ± 11 to 376 ± 24 Bq·m^−3^ (mean: 150 ± 19 Bq m^−3^), whereas indoor thoron concentrations ranged from below the Lower Limit of Detection (LLD) to 166 ± 10 Bq·m^−3^ (mean: 66 ± 8 Bq m^−3^), while radon and thoron concentrations in summer fell between 50 ± 11 and 305 ± 24 Bq·m^−3^ (mean 115 ± 18 Bq m^−3^) and from below the LLD to 122 ± 10 Bq m^−3^ (mean 48 ± 6 Bq·m^−3^), respectively. The annual average effective dose was estimated to be 3.7 ± 0.5 mSv yr^−1^. The soil-gas radon concentrations fell within the range from 1.07 ± 0.28 to 8.02 ± 0.65 kBq·m^−3^ (mean 3.07 ± 1.09 kBq·m^−3^). Finally, indoor radon maps were generated by ArcGIS software over a grid of 1 × 1 km^2^ using three different interpolation techniques. In grid cells where no data was observed, the arithmetic mean was used to predict a mean indoor radon concentration. Accordingly, inverse distance weighting (IDW) was proven to be more suitable for predicting mean indoor radon concentrations due to the lower mean absolute error (MAE) and root mean square error (RMSE). Meanwhile, the radiation health risk due to the residential exposure to radon and indoor gamma radiation exposure was also assessed.

## 1. Introduction

In general, people are exposed to ionizing radiation from various natural and artificial sources. Radon (^222^Rn), thoron (^220^Rn), and the progeny of both can be regarded as the largest contributor to the annual effective dose for the public in the world (50% of the total public dose) [1,2]; however, public exposure to ionizing radiation can be higher due to a new dose conversion factor [3]. Exposure to radon and its decay products is the second most common cause of lung cancer after tobacco smoking [4]. The health risks related to radon exposure primarily arise in indoor environments, while outdoor radon levels are generally low. The most important source of indoor radon is soil gas infiltration, and the intensity of this source relies on the composition of the ground, i.e., granite, tile, clay, etc. Soil gas infiltration is produced in mineral grains by the radioactive decay of ^226^Ra, emanated into the void spaces between the grains, transported by diffusion and advection/convection, and eventually exhaled from the soil into boreholes where it is detected. Moreover, cracks in concrete floors and walls, drainage pipes, connecting parts of buildings, heating, ventilation, and air conditioning ducts are the possible routes through which radon can enter into indoor environments [5].

Measuring indoor radon and thoron concentrations and radon mapping was considered for years and several papers were published on the topic around the world [6,7,8,9,10,11,12,13,14,15,16,17], including in many Iranian cities [18,19,20,21,22,23,24,25,26,27] to increase public awareness of environmental radioactivity and to predict radon-prone areas, which would help authorities with regard to the development of an appropriate strategy to reduce public exposure to radon and thoron. This reduced exposure would increase the quality of life and improve public long-term health. Due to the lack of data concerning indoor radon and thoron concentrations in houses in the city of Mashhad, an attempt was made to measure indoor radon and thoron concentrations in 78 houses to calculate the annual committed effective dose caused by the inhalation of radon and thoron. The measurements were taken during two seasons, summer (July–September 2019) and winter (December 2019–February 2020). The annual average radon and thoron concentrations were estimated by averaging measured concentrations during these periods. Subsequently, a radon map was produced using ArcGIS software and three different interpolation techniques, within a grid with the dimensions of 1 km × 1km. Meanwhile, the soil gas radon concentrations (as the major source of indoor radon) in different districts of Mashhad were also measured using a passive method based on CR-39 detectors during the summer when soil moisture and precipitation is low. Moreover, external exposure rates for terrestrial gamma radiation in Iran from 36 to 130 nGy h^−1^ with an average of 71 nGy h^−1^ have been reported [1,2]. In 2015, Sohrabi et al. also measured the indoors and outdoors gamma dose rates for about 1000 houses in 36 cities in Iran and the national mean background outdoor gamma dose rates were reported as being 70.2 nGy h^−1^ [28]. Therefore, to assess the radiation health risk, the public indoor doses from radon gas and indoor gamma radiation were compared and assessed.

## 2. Materials and Methods

### 2.1. Study Area

**Figure 1:**
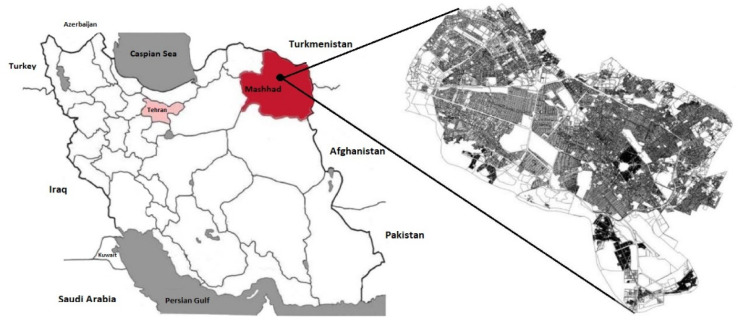
Location of the city of Mashhad in Razavi Khorasan Province, Iran.

Mashhad is the second largest metropolis in Iran and is the capital of Razavi Khorasan Province in northeastern Iran. It has an area of 351 km^2^ and its population is more than 3 million people according to the last census (Statistical Centre of Iran, 2016). It has witnessed rapid growth over the last two decades, mostly as a result of its economic, social, and religious attractions. The city is 985 m above sea level with the geographic coordinates of 36°17′45′′ N, 59°36′43′′ E. Geologically, the Kalaj mountains, which consist of granitic hills covered by silty deposits, are situated to the south of Mashhad, towards the northwest is Kale Ghaemabad that is comprised of more sandy soil, and in all other directions is a plateau with a mix of clay loam and soft sandy soils. Figure 1 shows the location of Mashhad in Iran. 

### 2.2. Measuring Techniques

Indoor radon and thoron concentrations are measured in the living rooms of houses at ground level. Regarding the recruited of participants, the priority was given to the older houses by selecting 3 to 5 dwellings from each district randomly depending on the size of the residential area. The majority of the houses examined were built 15 to 45 years ago using bricks composed of sand and cement along with cemented floors. The indoor measurements were conducted over a period of 90 days in total during the summer (July–September 2019) and winter (December 2019–February 2020). To determine the indoor radon and thoron concentrations, RADUET, a commercially available passive integrated radon–thoron discriminative detector, was used. These detectors consist of two diffusion chambers with different ventilation rates, and each chamber contains a CR-39 chip with the dimensions of 10 × 10 mm^2^ (RADUET, Radosys Ltd., Budapest, Hungary) for detecting the alpha particles emitted from radon and thoron as well as their progenies [29,30]. All detectors were hung at a height of 1–2 m above the ground using hard wire and positioned at least 20 cm away from any of the wall surfaces in the living rooms of the houses. 

In addition, a solid-state nuclear track detector (SSNTD), CR-39, was used to measure radon concentrations in soil gas. In this regard, a hole was dug in the soil of about 11 cm in diameter and 50–60 cm in depth. Then, a long PVC tube was fixed into the hole with the covered top end of the tube protruding from the ground by about 5 cm. At the bottom of each tube, a NRBP radon dosimeter [31] was placed for a period of 45 days between July and September 2019. 

After exposure, all detectors were wrapped in protective aluminum foil and returned for processing at the Institute. In the laboratory, they washed with distilled water and dried and then chemically etched. The etching condition for CR-39 was as follows: Solution: 6.0 M NaOH; Temperature: 90 °C; Time: 3 h. The track densities were counted using an optical transmission microscope and image analysis software. The calibration factors were determined as a result of exposure tests using radon and thoron calibration chambers at the Institute of Radiochemistry and Radioecology of the University of Pannonia, Hungary, and is comprehensively described in [30,31,32]. 

### 2.3. Annual Effective Dose, Excess Lifetime Cancer Risk (ELCR) and Lung Cancer Cases (LCC) Associated with Radon/Thoron Exposure

The annual committed effective doses originating from the inhalation of indoor radon or thoron were calculated using the following equation provided by [1]:

*E*_(Rn/Tn)_ = *C*_(Rn/Tn)_ × *F*_(Rn/Tn)_ × *t* × *K*_(Rn/Tn)_ (1)

where *E*_Rn/Tn_ denotes the annual committed effective dose from exposure to radon or thoron (mSv yr^−1^); *C*_Rn/Tn_ stands for the annual average radon or thoron concentrations in houses (Bq m^−3^); *F*_(Rn/Tn)_ represents the indoor equilibrium factors for radon or thoron and their respective progenies. The following values were provided by United Nations Scientific Committee on the Effects of Atomic Radiation (UNSCEAR) in 2000 [1]: *F* = 0.40 and *F* = 0.02 for radon and thoron, respectively; *t* is the number of hours spent inside annually (7000 h). Also, *K*_(Rn/Tn)_ denotes the following dose conversion factors recommended by UNSCEAR in 2000 [1]: *K*_Rn_ = 9 nSv and *K*_Tn_ = 40 nSv per unit of integrated radon and thoron concentrations (Bq h m^−3^), respectively.

In this survey, the average of the radon concentrations in the summer and winter represents the annual radon concentration. Furthermore, between December and February is regarded as the winter season when people tend to close windows because of the cold weather. The total exposure time was 90 days over the two seasons assessed. The Excess Lifetime Cancer Risk (*ELCR*) per 100,000 people was calculated using the following equation [33]:

*ELCR* = *E*_(Rn/Tn)_ × *D*_L_ × *R*_F_ (2)

where *D*_L_ represents the life expectancy, estimated to be 70 years; and *R*_F_ stands for the risk of fatal cancer per Sievert of 5.5 × 10^−2^ Sv^−1^ as recommended by International Commission on Radiological Protection (ICRP) Publication 103. Finally, the Lung Cancer Cases per year per million people (*LCC*) was estimated by using the risk factor lung cancer induction 18 × 10^−6^ mSv^−1^ and calculated by the following equation [34]:

*LCC* = *E*_(Rn/Tn)_ × 18 × 10^−6^. (3)

### 2.4. Statistical Analysis

The statistical analysis was carried out using IBM SPSS Statistics 21 (Armonk, NY, USA). The Kolmogorov-Smirnov test was applied to test the null hypothesis for the homogeneous distribution of the datasets. The Kruskal–Wallis non-parametric test with the Dunn’s post-hoc analysis was also used to test whether the samples originated from the same distribution based on the comparison of medians.

### 2.5. Radon Mapping and Cross-Validation

Radon mapping has great economic and social consequences; moreover, a high-resolution, accurate, and statistically powerful radon map is necessary to increase public awareness of environmental radioactivity and influence government policy with the purpose of reducing radon exposure in the general population. Depending on the datasets applied, two types of maps can be used: 1. Indoor Radon Maps which are based on indoor radon measurements (as applied in this study); and 2. Geogenic Radon Maps which are based on geological information [35]. The major merit of indoor radon maps is that radon concentrations are directly measured at the exposure point. 

In this study, an indoor radon map was generated by using ArcGIS software version 10.7 (GDi Esri Hungary Ltd., Budapest, Hungary) over a grid with the dimensions of 1 km × 1 km; moreover, three interpolation methods were tested: inverse distance weighting (IDW), ordinary kriging (OK), and Empirical Bayesian kriging (EBK). The arithmetic mean (AM) was used over grid cells with the dimensions of 1 km × 1 km to predict the mean indoor radon concentration on the ground floor of buildings in the grid cells where no data was available. It is important to keep in mind that radon maps are only a probabilistic tool to make policy decisions such as prioritization; they cannot be used to derive radon concentrations for an individual dwelling. By using some indexes, namely the mean absolute error (MAE), root mean square error (RMSE), root mean squared logarithmic error (RMSLE), percentage bias (PB), and coefficient of determination (R^2^), the accuracy of the different techniques was also examined, as presented in Equations (4)–(8):

(4) $$\mathrm{MAE}=\frac{1}{n}\sum_{i=1}^{n}\left|Z_{i}-X_{i}\right|$$

(5) $$\mathrm{RMSE}=\sqrt{\frac{1}{n}\sum_{i=1}^{n}{\left(Z_{i}-X_{i}\right)}^{2}}$$

(6) $$\mathrm{RMSLE}=\frac{1}{n}\sum_{i=1}^{n}{\left(log\left(Z_{i}+1\right)-log\left(X_{i}+1\right)\right)}^{2}$$

(7) $$\mathrm{PB}=100\frac{\sum_{i=1}^{n}\left(Z_{i}-X_{i}\right)}{\sum_{i=1}^{n}X_{i}}$$

(8) $$R^{2}=1-\frac{\sum_{i=1}^{n}{\left(Z_{i}-X_{i}\right)}^{2}}{\sum_{i=1}^{n}{\left(|X_{i}-\bar{X}|\right)}^{2}}$$

where *X*_i_ and *Z*_i_ denote the measured and predicted values in the location, *n* stands for the number of points in the validation group, and $\bar{X}$ represents the mean of *X*_i_. MAE and RMSE are often applied to assess the performance of models. The model fits properly if the aforementioned indicators approach zero when calculated. PB (%) is the mean of the tendency in larger/smaller predicted values than those observed [36]. R^2^ is the fit line explaining to which degree the model is going to fit to the dataset [37]. 

## 3. Results and Discussion

### 3.1. Activity Measurements

**Figure 2:**
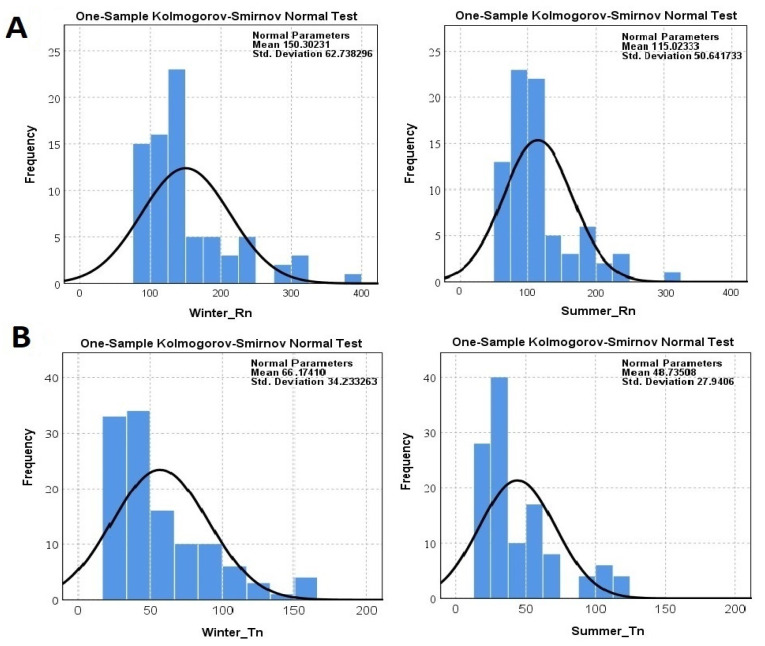
Normal Distribution of indoor (**A**) radon and (**B**) thoron concentrations (Bq m^−3^) over the two seasons at the ground level of dwellings examined in Mashhad.

**Figure 3:**
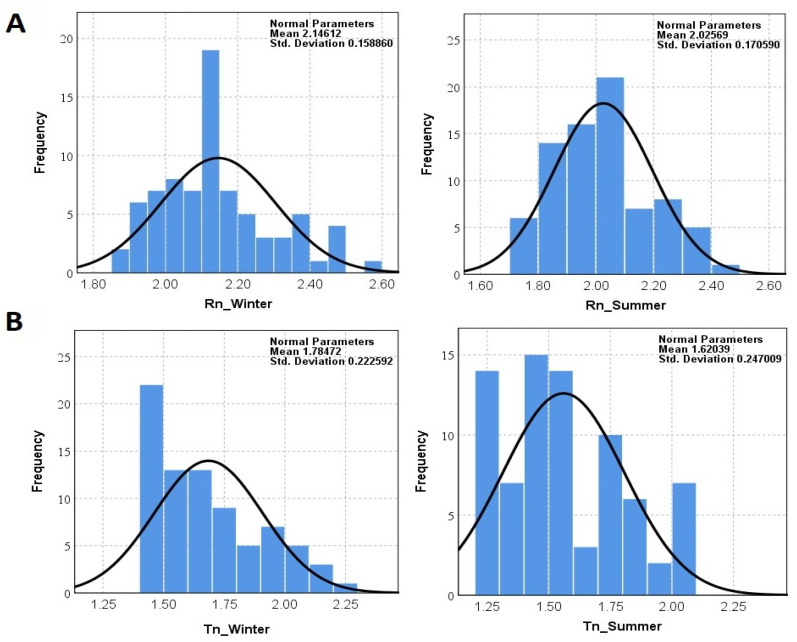
Log-Normal Distribution of indoor (**A**) radon and (**B**) thoron concentrations (Bq m^−3^) over the two seasons at the ground level of dwellings examined in Mashhad.

Within this study, the radon and thoron concentrations in 78 houses were surveyed over a total exposure time of 90 days (45 days in both the summer and the winter) by using a RADUET detector. The frequency distribution of the indoor radon and thoron for the 78 houses assessed in Mashhad over the two seasons are shown in Figure 2. In addition, a comparison among normal distribution and log-normal distribution of data is also illustrated in Figure 3. The indoor ^222^Rn and ^220^Rn concentrations in the winter ranged from 75 ± 11 to 376 ± 24 Bq·m^−3^ with a mean value of 150 ± 19 Bq·m^−3^ and from below the Lower Limit of Detection (LLD) to 166 ± 10 Bq·m^−3^ with a mean value of 66 ± 8 Bq m^−3^, respectively. In the case of the summer, the indoor ^222^Rn and ^220^Rn concentrations ranged from 50 ± 11 to 305 ± 24 Bq·m^−3^ with a mean value of 114 ± 18 Bq·m^−3^ and from below the LLD to 122 ± 10 Bq·m^−3^ with a mean value of 48 ± 6 Bq m^−3^, respectively. In addition, the annual average indoor ^222^Rn and ^220^Rn concentrations in the studied areas were 132 ± 19 Bq·m^−3^ and 58 ± 7 Bq m^−3^, respectively. The main source of indoor ^222^Rn originates from soil gas infiltration, building materials, and ventilation [4]. Meanwhile, during cold winters, residents use natural gas and close all vents, causing the radon accumulation found in houses.

World organizations such as ICRP, WHO, and U.S. Environmental Protection Agency (EPA) have recommended various guidelines for radon exposure [1,4,33,38,39]. The annual average indoor radon concentration is below the recommendation values (300 Bq m^−3^) provided by the ICRP in 2010. The results concerning the annual average radon concentration exceed the action level (100 Bq m^−3^) recommended by the WHO in 2009. When compared to the worldwide geometric mean (GM) of 37 Bq·m^−3^ (geometric standard deviation (GSD) = 2.2) reported by UNSCEAR in 2000, the indoor radon in the city of Mashhad is almost 4 times (139.68 Bq m^−3^) and approximately 3 times (105.8 Bq m^−3^) higher than the world average in the winter and summer, respectively. It was also found that during the winter and summer, the indoor radon concentrations in 31% and 20% of the dwellings were higher than the reference level of 148 Bq·m^−3^ recommended by the US EPA in 2003.

**Figure 4:**
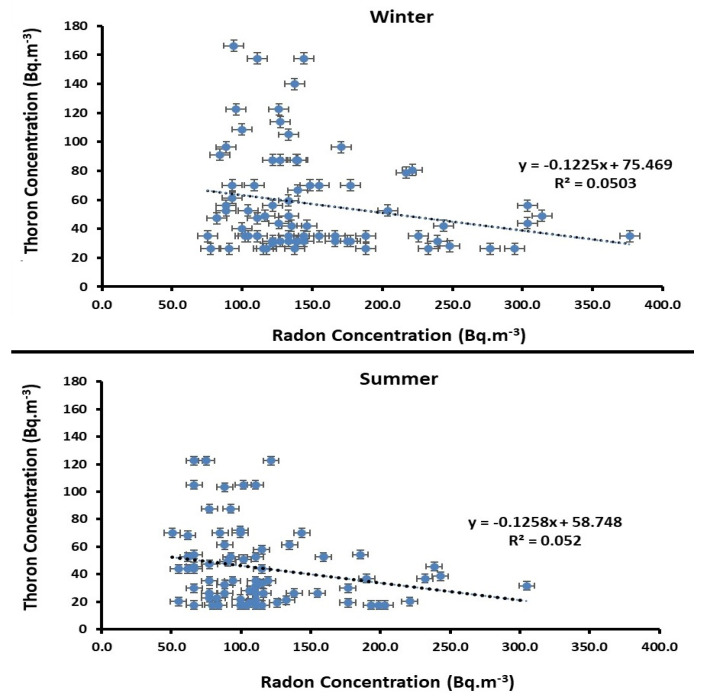
Correlation between the indoor radon and thoron concentrations of the 78 houses examined in Mashhad over two seasons.

The graph in Figure 4 shows the correlation between indoor ^222^Rn and ^220^Rn concentrations for the dwellings examined in Mashhad. Regarding the relationship between radon and thoron concentrations, no clear and strong correlation between was observed and thoron concentrations could not be predicted from widely available information concerning radon. However, indoor radon and thoron concentration might directly depend on the activity of ^226^Ra and ^232^Th (^228^Th) in building materials, ground is the main entry path of radon at dwellings; therefore, it could say that the content of both ^222^Rn and ^220^Rn depends on the building materials and soil composition.

**Figure 5:**
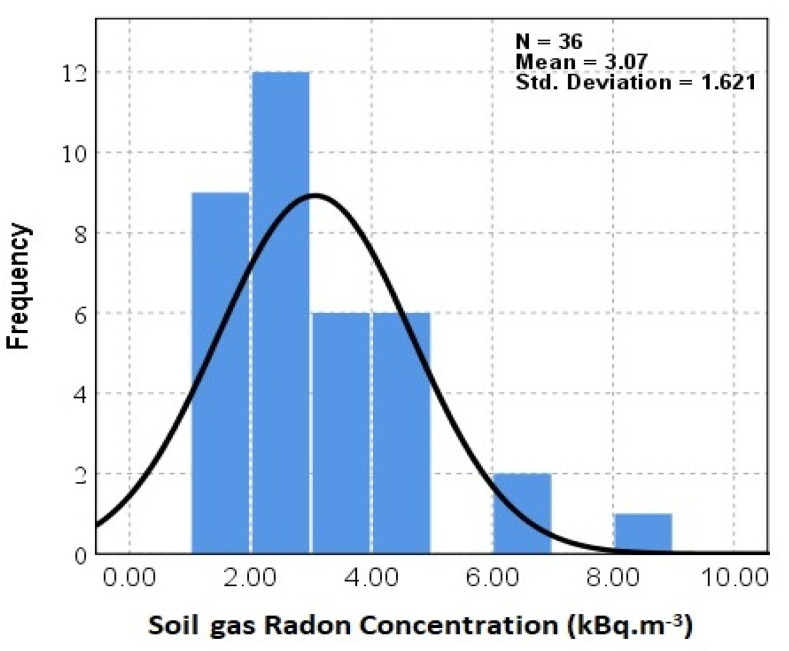
The soil-gas radon concentrations (kBq m^−3^) in the studied area.

**Figure 6:**
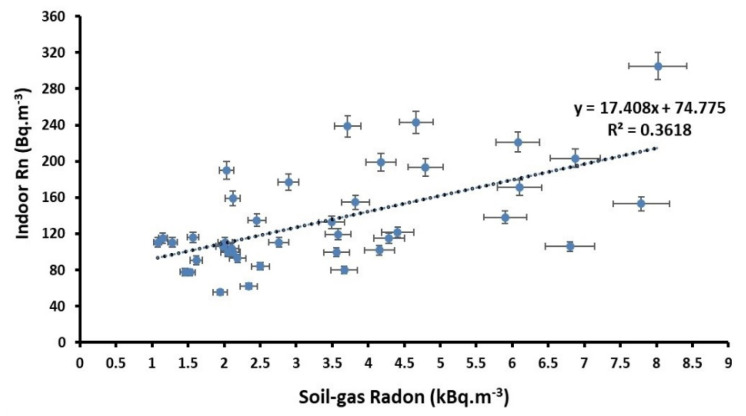
The correlation between indoor radon and soil-gas radon concentrations.

As previously discussed, the main source of indoor ^222^Rn originates from soil gas infiltration, ^222^Rn concentrations in the soil gas of different districts in Mashhad were measured by using a passive method based on CR-39 detectors in the summer when soil moisture and precipitation are low. In order to determine soil gas radon concentrations, only 36 NRPB dosimeters were retrieved from where they were set up, while the remaining 6 dosimeters were considered lost. Figure 5 shows a histogram of soil gas radon concentrations in Mashhad during the summer. The soil gas radon concentrations recorded in the studied area fell within the range of 1.07 ± 0.28 to 8.02 ± 0.65 kBq·m^−3^ with a mean value of 3.07 ± 1.09 kBq m^−3^. As is shown in Figure 4, the activity concentrations of ^222^Rn vary from location to location, possibly because of the physic geological properties of the types of soil studied, topographic differences, as well as geomorphology and meteorological conditions of the region. The average radon concentrations in both soil gas and indoor environments are approximately the minimum and maximum values in the same region, respectively. Moreover, the correlation between indoor radon and soil gas radon concentrations for the districts studied is shown in Figure 6. The correlation analysis yielded a positive correlation (R^2^ = 0.361) between average indoor radon and soil-gas radon concentrations.

The normality distribution of data was checked using the Kolmogorov–Smirnov test. Considering the normality assumption in the null hypothesis of the Kolmogorov–Smirnov test, the probability value (*p*-value) in all tests was less than 1%; therefore, the normality distribution of radon and thoron concentrations in any of the following subfactors was rejected. In this study, by applying the Kruskal–Wallis nonparametric test with Dunn’s post hoc analysis, the null hypothesis, due to the absence of a statistically significant difference in the average gas concentration, was rejected; therefore, the season and type of gas affect the gas concentration (*p*-value < 0.05). The difference in radon concentrations between well-ventilated and poorly ventilated dwellings was statistically significant (*p* < 0.05). It was assumed that houses with natural ventilation are poorly ventilated and houses mechanical ventilation systems are well-ventilated houses. The finding indicates that the radon concentration is lower for well-ventilated dwellings compared to poorly ventilated ones. The results of this study are consistent with others that have been conducted concerning this topic [40,41,42]. Because the level of indoor radon concentration depends on the degree of indoor ventilation, moreover, in well-ventilated dwellings, radon can easily escape and does not accumulate inside, meaning indoor radon concentrations are less high in well-ventilated dwellings compared to in poorly ventilated ones [40].

Table 1 shows descriptive statistics that resulted from the measurement of the indoor radon and thoron concentrations in the 78 houses studied in Mashhad during the two seasons considered. Furthermore, the results reveal a seasonal variation in indoor radon and thoron concentrations, which were higher in the winter than in the summer. This is because the doors and windows of dwellings remain closed most of the time in the winter compared to in the summer, hence ventilation is poorer in the winter. The ratio of winter to summer concerning indoor radon and thoron concentrations was also established for all 78 dwellings studied. This ratio of indoor radon concentrations ranged from 1.23 to 1.48 with an average value of 1.31. With regard to the indoor thoron concentration, the average of this ratio was similar, at 1.36. The reason of heterogeneous behavior of seasonal variations in ^222^Rn and ^220^Rn concentrations might be that the source of ^220^Rn is mainly limited to the concentration of ^232^Th (^228^Th) in building materials, while in case of radon, the ground’s concentration is additionally considered. Therefore, in summer due to a high air exchange rate, e.g., using a ventilator or opening windows, the concentration of both ^220^Rn and ^222^Rn goes down, while in winter since the air exchange rate is lower than summer, the concentration of ^220^Rn and ^222^Rn build up but as the source of indoor ^222^Rn is both ground and building material rather than the only source of ^220^Rn as building materials, the seasonal change of indoor thoron concentration is less than that of indoor radon.

A comparison of radon concentration in the soil gas under investigation with those reported in other countries is also given in Table 2. It can clearly be seen that the radon concentration in soil samples from the Sri Ganganagar district and the northern state of Rajasthan in India, the city of Najaf in Iraq, and Yemen are in close agreement with the present work. It can be concluded that the soil in Mashhad is suitable for construction without posing any health hazards.

### 3.2. Radiation Dose and Risk Assessment

In this study, the average of the radon concentrations in the summer and winter was assumed to be the annual average radon concentration. The corresponding annual effective dose from the inhalation of radon and thoron was calculated as 3.7 ± 0.5 mSv yr^−1^. The committed effective dose from indoor radon and thoron was found to vary from 2.11 to 9.73 mSv yr^−1^ with a mean value of 4.22 mSv yr^−1^ for the winter, and 1.51 to 7.92 mSv yr^−1^ with a mean value of 3.14 mSv yr^−1^ for the summer. It can be seen that the committed effective doses were higher in the winter compared to the summer. The excess lifetime cancer risk and lung cancer cases per year per million people were also calculated to be 14.1 and 65.4, respectively.

According to the WHO in 2009, the risk of lung cancer increases by 16% per a 100 Bq·m^−3^ increase in radon concentration (long-time average) [4]. Therefore, the dose-response relation is linear, i.e., the risk of lung cancer is proportional to radon exposure [4]. Nevertheless, based on a report produced by the Ministry of Health’s Center for Disease Management in Iran, cancer is the third most significant cause of death after road traffic accidents and cardiovascular mortality. In 2019, Roshandel et al. reported that the age-standardized rates (ASR) of lung cancer were 127 and 52.1 per 100,000 Iranian males and females, respectively [52]. In the case of Razavi Khorasan Province, the ASR was 121.2 and 54.0 per 100,000 Iranian males and females, respectively. Therefore, the annual average excess risk due to radon inhalation in Mashhad is 14/100,000, i.e., less than the age-standardized death rate from cancer in Mashhad. Hence, indoor radon exposure is responsible for approximately 12% of lung cancer deaths in this city, which is close to the estimates by WHO in 2009 of the worldwide proportion of lung cancer due to radon (3–14%).

Given the annual average excess risk values by comparing local radiological risks with national cancer incidence data, it can be concluded that the local risks are raised but are not necessarily representative of the city as a whole. This radiation risk assessment should be considered with caution as the radon measurements are not sufficiently representative of the investigated area; moreover, calculations made using ICRP data only provide a broad overview of the risk and comparison with the national cancer incidence rate. Therefore, extensive measurements are needed for a reliable comparison.

It is also essential to measure the amount of natural radiation in each area as this can determine the suitability of the environment for a healthy lifestyle. The indoor and outdoor gamma exposure rates in the air 1 m above the ground from terrestrial radionuclides and cosmic rays in Mashhad are 155.73 ± 13.92 nGy h^−1^ and 126.15 ± 15.66 nGy h^−1^, respectively [28]. Using a determined conversion factor as 0.7 Sv Gy^−1^, converting the absorbed doses to effective doses [1], the annual indoor and outdoor effective dose rates of the public from gamma exposure were found to be 0.95 ± 0.08 and 0.77 ± 0.09 mSv yr^−1^. Therefore, by comparing these values with the corresponding annual effective doses from the inhalation of radon and thoron (3.7 ± 0.5 mSv yr^−1^), it could be concluded that most of the doses received indoors in the dwellings studied in Mashhad city are from the inhalation of radon and thoron (about 79 % of the total dose). A comparison of the indoor radon concentration and radiation risk assessment under investigation, with those reported in other Iranian cities also provided in Table 3.

### 3.3. Spatial Distribution Map of Indoor Radon Concentrations

The Distance Weighting (IDW) and Ordinary Kriging (OK) techniques, known as Kriging techniques, depend on the distance between two points, namely those of observation and estimation in the interpolation. IDW weighted the contribution of the observed points on the estimated interpolation with regard to this distance alone. On the other hand, OK also considers the correlation between the points and forms an initial function, i.e., covariance or variogram, which can iteratively be updated.

**Figure 7:**
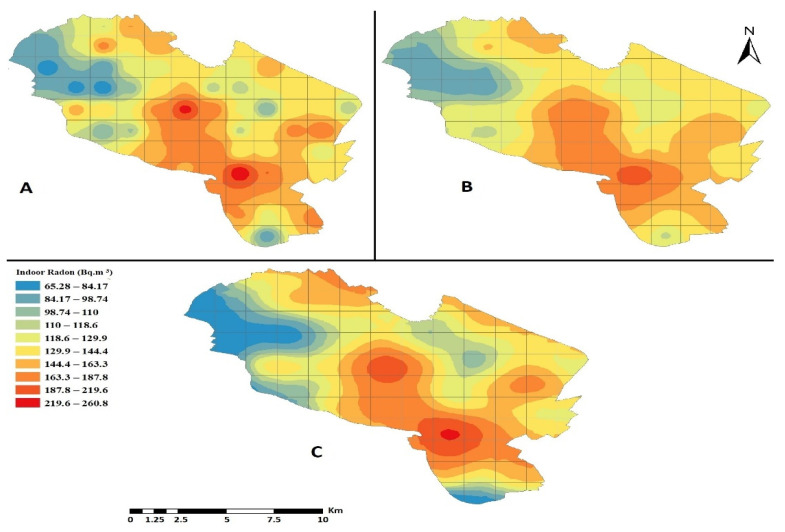
Predicted Indoor Radon Map of Mashhad dwellings over a grid with the dimensions of 1 km × 1 km using the (**A**) Inverse distance weighting, (**B**) Empirical Bayesian Kriging, and (**C**) Ordinary Kriging interpolation techniques.

The spatial distribution map of indoor radon concentrations in Mashhad dwellings were plotted in Figure 7 by various interpolation techniques, e.g., IDW, OK, and Empirical Bayesian Kriging (EBK) over a grid with the dimensions of 1 km × 1 km using ArcGIS software version 10.7. Accordingly, radon concentrations were lower than standard values in eastern residential areas and were higher in central as well as southern districts. Nevertheless, when the spatial autocorrelation between cells was considered, predictions about radon concentrations using different methods range from 65 to 260 Bq m^−3^. These values may be more realistic and similar to average values found in some dwellings in the region.

Moreover, the accuracies of the various techniques applied according to five indicators are given in Table 4. IDW, which predicts unknown values using known values concerning their distance, was proven to be more suitable for predicting mean indoor radon concentrations over grids with the dimensions of 1 km × 1 km (i.e., arithmetic mean, ground floor), due to the lower MAE and RMSLE values of 28.159 and 0.01210, respectively, in addition to a lower bias, 20.069 to be exact. However, all mentioned models have a tendency to overestimate bias (PB > 0). In addition, the model with the higher R^2^ is IDW, which indicates that this model fits the data better.

## 4. Conclusions

To estimate the impact of indoor radon and thoron on residentials as well as develop and implement the most economical method to reduce radon exposure using a radon map, this paper presents the measured indoor radon and thoron concentrations in 78 dwellings as well as soil gas radon concentrations in different districts of Mashhad, Iran during summer and winter. As the average of the radon concentrations in the summer and winter were assumed to be the annual average radon concentration in this study, the annual average indoor radon and thoron concentrations were calculated as being 132 ± 19 and 58 ± 7 Bq m^−3^, respectively. Soil gas radon concentrations also ranged from 1.07 ± 0.28 to 8.02 ± 0.65 kBq·m^−3^ with a mean value of 3.07 ± 1.09 kBq·m^−3^ during the summer.

The corresponding annual effective dose from the inhalation of radon and thoron was calculated as being 3.7 ± 0.5 mSv yr^−1^. Subsequently, the excess lifetime cancer risk was calculated as 14.13. Hence, exposure to indoor radon is responsible for approximately 12% of lung cancer deaths in Mashhad, which is close to the WHO estimates of the worldwide proportion of lung cancer due to radon (3–14%). By comparing the annual indoor effective dose rate from gamma exposure with the annual effective dose from the inhalation of radon and thoron, it was concluded that most of the dose received inside the dwellings studied in Mashhad, approximately 79% of the total dose, originates from the inhalation of radon and thoron.

Since high-risk areas can be recognized on radon maps, which are useful for targeting landlords and the building industry, an indoor radon map was generated by using ArcGIS software over a grid with the dimensions of 1 km × 1 km using three interpolation techniques. The arithmetic mean was used over the grid cells to predict a mean indoor radon concentration on the ground-floor level of buildings in the grid cells where no data was available. The IDW technique was proven to be most suitable one for predicting mean indoor radon concentrations over grids with the dimensions of 1 km × 1 km.

In addition to the results and given the significant health impacts of radon and thoron, it is hoped that both radon and thorn gases will be studied more seriously in Iran and that these techniques as well as complementary procedures will be used to minimize its concentration. It is recommended that radon gas concentrations should be measured in all regions of the country by numerous devices supplied by the Atomic Energy Organization of Iran (AEOI). As a result, it would be possible to compile a radon map of Iran to estimate the concentration and number of radon-induced incidences of cancer, as well as decide how to distribute the population. This would aid to reduce the number of cases of lung cancer and other radon-induced human health problems.

## Figures and Tables

**Table 1:** Basic Statistics of indoor and soil gas ^222^Rn and ^220^Rn concentrations in samples from Mashhad.

Season	Parameter	A.M ^1^	G.M ^2^	S.D ^3^	Min	Max	Winter/Summer Ratio (Mean)	
							^222^Rn	^220^Rn
Summer	Indoor air radon (Bq m^−3^)	115.02	105.8	50.64	50.8	305.2	1.31	1.36
	Indoor air thoron (Bq m^−3^)	48.73	37.4	27.95	<LLD	122.5		
	Soil-gas radon (kBq m^−3^)	3.07	2.71	1.621	1.078	8.021		
Winter	Indoor air radon (Bq m^−3^)	150.3	139.68	62.74	75.3	376.6		
	Indoor air thoron (Bq m^−3^)	66.17	49.41	34.24	<LLD	166.3		

^1^ A.M = Arithmetical mean, ^2^ G.M = Geometrical mean, ^3^ S.D = Standard deviation.

**Table 2:** Comparison of soil-gas radon concentrations under investigation with those in other countries using different methods and sampling depths.

Region	Radon in Soil-Gas (KBq m^−3^)	Measurement Method	Sampling Depth (cm)	Reference
Bǎita-Stei, Romania	5.5–512	Lucas Cell	40–80	[43]
Bolsena, Italy	7–176	RAD 7	60–70	[14]
Bulgaria	3–97	AlphaGuard	100	[44]
Hungary	1–47.1	RAD 7	80	[45]
Najaf, Iraq	0.009–9.29	RAD 7	5–60	[46]
Rajasthan, India	0.94–10.05	RAD 7	100	[47]
Sharr-Korabi, Kosovo	0.295–32	SSNTDs (CR-39)	80	[48]
Slovenia	0.9–32.9	AlphaGuard	100	[49]
Sri Ganganagar, India	0.9–10.10	RAD 7	10–100	[50]
Yemen	0.15–13.56	SSNTDs (CR-39)	0–150	[51]
Mashhad, Iran	1.07–8.02	SSNTDs (CR-39)	50–60	Present study

**Table 3:** Studies on indoor radon concentration (Bq m^−3^) and radiation health risk in various Iranian cities.

Region	Number of Dwelling	Mean Radon Concentration (SD ^1^)	Mean Effective Dose (mSv yr^−1^)	*ELCR* ^2^	*LCC*^3^ × 10^−6^	Excessive Rate (%)	Reference
Isfahan	51	28.57 (39.38)	0.72	2.7 × 10^−1^	12.96	4% > 100 Bq m^−3^	[26]
Lahijan	400	163 (57)	3.43	1.3 × 10^−2^	61.74	In most dwellings > 100 Bq m^−3^	[19]
Mashhad	148	31.9	(0.25–3.78)	-	-	5.3% of apartments > 100 Bq m^−3^	[22]
Qom	123	95.83	2.41	9.2 × 10^−3^	43.38	24.3% > 100 Bq m^−3^	[23]
Ramsar	500	Autumn: 355 Winter: 476	Autumn: 8.95 Winter: 12	3.44 × 10^−2^ 4.6 × 10^−2^	161.11 216	-	[18]
Shiraz	185	57.6 (33.06)	1.45	5.6 × 10^−3^	26.1	5.4% > 100 Bq m^−3^	[24]
Tehran	30	104	2.62	1 × 10^−2^	47.16	38% > 100 Bq m^−3^	[25]
Yazd	84	137.4 (149.5)	3.46	1.3 × 10^−2^	62.28	30% of basements> 148 Bq m^−3^	[21]
Mashhad	78	Summer: 115(51) Winter: 150 (62)	Summer: 3.1 Winter: 4.2	(12.3 × 10^−3^) (15.9 × 10^−3^)	(56.7) (74.1)	Summer: 20% > 148 Bq m^−3^ Winter: 31% > 148 Bq m^−3^	Current study

^1^ SD= Standard deviation, ^2^ ELCR= Excess Lifetime Cancer Risk, ^3^ LCC= Lung Cancer Cases.

**Table 4:** Summary of the cross-validation results.

Method	MAE ^1^	RMSE ^2^	RMSLE ^3^	PB ^4^	R^2^
Inverse distance weighting	28.159	34.931	0.01210	20.069	0.234
Empirical Bayesian Kriging	28.235	35.148	0.01218	20.123	0.224
Ordinary Kriging	28.424	36.364	0.01346	20.268	0.169

^1^ MAE = mean absolute error, ^2^ RMSE = root mean square error, ^3^ RMSLE = root mean squared logarithmic error, ^4^ PB= percentage bias.

## Data Availability

The data presented in this study are available on request from the corresponding author.
